# Treatment of Pyorrhea

**Published:** 1907-09-15

**Authors:** Grace Pearl Rogers

**Affiliations:** Detroit


### TREATMENT OF PYORRHEA.

BY GRACE PEARL ROGERS, D. D. S., DETROIT.

The subjects demanding the dentist's attention today are numerous and varied, but, among them all there is not one of more vital importance than the treatment of pyorrhea. The object of our calling is to save the human teeth, and this purpose we cannot attain by the most perfect and artistic repairing of the crowns, without preserving the foundation upon which the crowns rest; for a perfect tooth is of no value to a patient if its attachment has been destroyed by disease.

By common usage the name Pyorrhea Alveolaris has come to designate a variety of conditions, however, the term is at fault, like many others of our profession. It might not be an unwise plan to have a committee appointed to revise the nomenclature of our profession. If this were done much of the time which is consumed in discussing the applicability of certain terms would be put to better use. The energy spent at dental meetings in the last twenty-five years discussing the use and abuse of the name pyorrhea, in suggesting substitutes for the same, and in arguing for a local or constitutional cause, would have been sufficient to cure many cases of the disease. So, for the sake of simplification, let it be understood that when in this article the term pyorrhea is used, I mean any condition which has resulted in the destruction of peridental membrane. If any portion of the membrane is destroyed, a pocket is formed which, whether it contains pus or not, is of serious importance.

Beginning about the middle of the 19th century, with the work done by Dr. John M. Riggs, we find that there have always been, and still are, some few in the profession who have not only claimed to cure, but have cured many cases of pyorrhea. Why has the cure not been universal, and why do so many dentists say the disease is incurable, and give their patients no hope when they present themselves with diseased tissues and loose teeth? In spite of the fact that articles almost innumerable have been written upon this subject, and discussed pro and con, teeth are being lost from this malady in deplorably large numbers every day.

With a few notable exceptions, the members of our profession have been talking and not acting. The majority have not been able to recognize the disease in its incipient stage, consequently the trouble has progressed unmolested until some day a very loose tooth distresses a patient, and it is extracted by his dentist, who shakes his head and gives the patient the following consolation.

"You have Pyorrhea Alveolaris, and will have to lose all your teeth. It is caused by uric acid in the system, and there is no cure for it. You will be wearing plates before you are fifty years old." Perhaps another dentist would have advised treatment. This consisting in a careless scaling of the teeth with inefficient instruments and syringing out the pockets with an antiseptic. Such a treatment is better than none, but it will not *cure* the trouble. Some more progressive man would have treated the case in a more careful way until the mouth was put in a healthy condition. He would not have extracted the loose tooth until he had proven to his own satisfaction and to that of his patient, that to save it was impossible. Some teeth which seem hopelessly loose, often respond in a surprising manner to the proper treatment. Therefore, it is always best to at least try to save most teeth before using the last resort—*extraction*.

The moral effect this has upon the patient is most important, even though he should eventually lose the tooth. The *dentist himself puts the estimate of value upon his patient's teeth*, and if the extraction of a tooth means nothing to him, it will mean no more to the patient. If on the other hand he puts forth every effort to save a tooth, even though diseased and teaches his patient the importance of preserving the natural teeth, the latter will appreciate it and take better care of his remaining teeth. Ten years from now I believe that the dentist who extracts a tooth because it is affected with pyorrhea without first using every possible means of saving it, will be placed in the same class with the man who extracts a tooth because it is abscessed, without attempting to treat the abscess. There is no *short road* to success in the treatment of pyorrhea, such as the alkalithia people and compounders of various mouth washes would have us believe. We cannot sit in the shade with Bill Nye and Dr. C. M. Wright any longer, but must get out in the sun and work.

While the profession is divided on the subject of the *cause* of the disease under consideration, nothing can be accomplished by more argument. There are a few things which we know *will* cause pyorrhea under proper conditions, and in so far as we know, we should act. It is for the men connected with our colleges, who have the time to prove scientifically and practically, what influence constitutional disturbances have upon this trouble, and vice versa, we can add to their effort the result of our observations,but no man in general practice handles enough cases to make any decided statements either way. It means years of research work added to what has already been done before we can reach positive conclusions.

I believe that every dentist who has been successful in the local treatment of pyorrhea, has found that that treatment alone was sufficient. Dr. Riggs, who with the crude instruments he must have had to work with, cured 90 per cent of all the cases coming under his care. Dr. D. D. Smith cures even a larger per cent. The works of these men stand out as beacon lights in the history of dentistry to encourage us in our attempts to treat pyorrhea. The cures made were complete, and their treatment entirely local. It is the belief of many dentists who are successful in the treatment of this malady, that if the gum tissue be kept absolutely free from local irritation, no condition of the system can bring about pyorrhea. This has been *proven* by the Oral Prophylaxis treatment as practiced by Dr. Smith and others. Those of us then who cannot cure pyorrhea can at least *prevent* it, and that then is our duty. However, until we are converted to the need of preventing the disease of the soft tissues of the mouth, or at least checking pyorrhea, in its incipiency we will have to treat this disease in its advanced stages, which is one of the most difficult operations a dentist has to perform.

While this disease may not be fully understood, yet a classification of the knowledge we have upon it may be of value. The causes of diseases are divided into:

(a) Indirect or Predisposing.
(b) Direct or Exciting.

### PYORRHEA.

1. Predisposing Causes.—(a) Mal-occlusion.
  - (a) Misuse.
  - (b) Nonuse.
  - (c) Overuse.
  - (d) Making the prevention of accumulations difficult.
  - (e) Lack of development of tissues surrounding roots.

(B) Tooth Extraction:
  - (a) Making the prevention of accumulations difficult.
  - (b) Mal-occlusion.

(C) Low Vitality.
  - (a) Lessens resistance of soft tissues to irritants of any kind.

(D) Caries.—Direct Causes
  - (a) Mechanical—Bands, Crowns, Fillings, Plates, Tartar, Clamps, Ligatures.
  - (b) Chemical—Soft deposits, Slippery Coating.
  - (c) Infectious—Bacteria.

I do not wish to leave the impression that this covers the etiology of Pyorrhea, but rather to place before you conditions which are known to produce this disease, and to emphasize the importance of using the knowledge that we have.

Let us suppose that we are not able to cure every case that comes to us, is it not worth our while to at least cure those we can?

After reading the literature on this subject, and obtaining much information from the various schools in our country I came to the conclusion that so far as the treatment of pyorrhea was concerned, "there is nothing new under the sun." I therefore shall not attempt to give you anything new, but merely to give the method of treatment in detail, and to lay emphasis upon certain parts of it which have, I believe, been considered unimportant by the men who have not been successful in the work. The general impression has been that the secret of success lay in a certain set of instruments, or in an especial medicament, while in fact the results of the work depends upon the thoroughness with which it is done. Every source of irritation must be removed, and if you can accomplish this with three or four instruments, each one taken from a different set, the cure will be as complete as the one accomplished by another dentist who used the 27 instruments comprising the Younger set, The therapeutic remedy applied is merely an *aid* to Nature in repair of tissue, and even the most efficient will accomplish little unless all irritants have been removed.

I shall divide the subject into: The Curative and Preventative phases.

In the treatment of a disease, if a physician does not know the cause, he treats the symptoms as they arise. If you can locate the direct cause of the disturbance around a root, then remove it; if not, then you are left to treat the symptoms. You have an inflammation (with or without pus present) to relieve. If pus germs are present they must be destroyed, all source of irritation must be removed, and as nearly as possible the parts must be kept clean and at rest. If the teeth to be operated on are very loose and it is possible to ligature them, do so; for if the teeth are held firm it will facilitate your work and will make the operation more comfortable for your patient. Permanent splints should be supplied when necessary.

Surgery of the most intricate kind is involved in the Curative phase of the treatment, and it is necessary at first to have our field of operation as nearly aseptic as is possible. There is no antiseptic of which we have knowledge which will accomplish much in a mouth where the teeth are partially covered with bacterial plaques and soft deposits, as is usually the condition in a Pyorrhetic mouth. Consequently we must obey the first rule of antiseptic surgery, to first remove all that we can mechanically. In regard to the teeth this would mean the careful removal of all soft deposits, tartar, stains and bacterial plaques from the exposed tooth surfaces. This should be thouroghly done, in a way which will best accomplish the required results. If this work is then followed by the sprays recommended by Dr. Ferris, and introduced to us by Dr. White, we would have an area in which to operate that would be very nearly aseptic. This preparatory work will require some little time, and you may not be able to do more than this at the first sitting. There are several advantages, too, in not trying to do more.

1st. A patient usually dreads the treatments, and if you cause him no discomfort the first time, his fear will be essened.

2nd. The gum tissue will respond to the removal of external irritation, and be less sensitive at subsequent treatments.

3rd. You will have a much more agreeable atmosphere in which to work, for there will be no odors emanating from soft accumulations.

4th. You will have less hemorrhage, and a bloody operation is never desirable either for you or your patient.

Of course, if your patient is suffering, relief from pain is the first requisite; if not then you may use your own judgment in manner of treatment.

You should not overtax your patient's strength, because much benefit may be lost; for any diseased organ needs back of it a healthy constitution to aid in its recovery, and anything which tends to undermine the health, such as a nervous strain, tends to retard repair.

You should impress upon your patient's mind most emphatically the absolute necessity of systematic and thorough personal care of the mouth and teeth. The importance of this cannot be overestimated, and neither you nor your patient can afford to overlook it. Without your patient's co-operation, you cannot produce a healthy mouth. It is impossible; for it requires but a few days of neglect in order that many substances may accumulate on and around the teeth, which are irritating to the soft tissues of the mouth. If there is a source of constant irritation, how can a diseased organ be improved? In this connection the question of tooth brushes, tooth picks and dental floss, comes up for consideration. No matter what interest a patient may have in his own case, he cannot accomplish much unless he has the proper instruments with which to operate. There are brushes made which are adapted to almost every condition we may chance to meet. Teeth standing alone, bridge work, long and short crowned teeth, regulating appliances, all these demand different brushes in order to procure the best results. If but one brush is used, and that three or four times a day, the bristles become soft and discolored, which of course is objectionable, and the necessity of more than one brush of its kind, very evident. The use of tooth picks should be discouraged, excepting in rare cases. It is a vulgar habit when indulged in anywhere, except in the privacy of one's own room. Its object is the removal of food particles, while in fact only the large ones can be removed by its use. The numerous small ones are only pushed further in between the teeth. These latter can be removed by using dental floss, which is an indispensable article in the personal care of the oral cavity. I would rather that a patient of mine use dental floss intelligently once each day, (and that just before retiring at night), than a tooth pick after every meal. The floss is not only useful in removing food particles from between the teeth, but if properly manipulated will help to break up the bacterial plaques, which otherwise remain undisturbed on the approximal tooth surfaces. When you have gum recession, and the contact points are not too close the unwaxed ribbon floss (fine) is most effective, while the waxed (standard flat) is satisfactory in ordinary cases.

See to it that your patient has the proper quality and quantity of materials with which to work, and that he understands how to use them. He might brush his teeth five times each day unintelligently and not accomplish as much as some other person who used his tooth brush only once each day, but then in an intelligent way.

Before the patient is dismissed from the first sitting he should be given detailed instructions in the care of his mouth and teeth. Do not take for granted that he understands anything about it, but give him a very thorough lesson. Oftentimes I believe we make the mistake of neglecting to explain some very simple thing, thinking it unnecessary, when in this work it is the little things which are most important.

There are but few patients, and I may add a very small number of dentists who know how to care for their mouths. An intelligent person knows that he ought to keep his teeth clean, and he may do so to the best of his knowledge, but that part of his education has usually been neglected. It is our duty to study each case, point out the surfaces which are neglected, and give the necessary instruction. This is compulsory in the treatment of pyorrhea, for if you neglect this important part of your treatment the results of your work will be unsatisfactory. Where there has been much destruction or recession of tissue the teeth are especially hard to care for, since there is so much tooth surface exposed. Almost every mouth will offer some problem for solution in the matter of personal care, and these problems *must* be solved by the dentist. To some of you this question may seem of minor importance, but speaking from my own observation I believe that a very large number of failures in the treatment of pyorrhea are due to neglecting this apparently minor point.

You should be familiar with the constituents of any powder or mouth wash you recommend; for they are used so frequently that much harm may follow if any injurious ingredient is contained. An astringent mouth wash is very helpful while treating pyorrhea.

About forty-eight hours should elapse before a second sitting is given, and then you must use your own judgment as to the location and number of teeth operated on. If the patient has been faithful and intelligent in his care you will not be able to see any deposits on or about his teeth; therefore an efficient spray is all that is necessary in order to prepare for the operation. The difficult part of your work is now to begin, viz, the scaling of the roots guided only by the sense of touch. As you all know, the tactile sense is located in the fingers, and while a delicate sense of touch is natural to some, yet any one by experience and practice can develop what faculty he may have in that direction. One should be able to recognize live bone, dead bone, tooth and deposit solely by the sense of touch. Without this skill your work necessarily cannot be thorough, no matter how conscientious you may be.

This skill cannot be obtained outside the mouth, hence the need of education in this field of work while in college. No student should be allowed to graduate who has not thoroughly scaled a sufficient number of teeth to make him reasonably skillful, at least so that he may be able to meet with some degree of success when in practice.

There are many scalers on the market, and no doubt each one has its place of usefulness. However, no matter how many scalers you may possess, there are always favorites which prove themselves most valuable to you. To some men certain instruments are indespensible, while to others these same instruments are of little value. It is best, however, to have a large number from which to choose. Large, clumsy scalers are objectionable for two reasons: gum tissue is unnecessarily lacerated, and with them you can only recognize the large deposits. The smaller points are much more preferable and useful. The Smith and Ferris-Tompkins files are indespensable, for with them one can detect the most minute granules. Excepting in rare instances, it is not necessary to use a local anesthetic. I have not yet found it necessary in any case. When the gums are anesthetized the operator is unconsciously less careful, gum tissue is lacerated unnecessarily, and there is as a result just that much more tissue for nature to repair. I believe too that an anesthetic interferes with the healing process. If, in working in a pocket, one confines himself to the area bounded by the walls of the pocket he will not cause the patient pain.

Every trace of necrosed bone must be removed, as well as all deposits of any form or kind. Carefully rinse in an antiseptic, and wipe instruments whenever you remove them from the mouth. If you are not careful to do this you are apt to either carry back into the pocket material you have removed, or you may infect another pocket with this material. Be sure to remove everything from the pockets which you have from the tooth or process. Carefully syringe out pockets with warm phenol sodique and then medicate with an efficient antiseptic. The four antiseptics most universally used are: 25 per cent trichlor acetic acid, lactic acid, full strength, aromatic sulphuric acid 25 per cent, zinc chloride 50 per cent. Any one of these will prove satisfactory if your work has been thorough. An orange wood applicator is best for applying the medication, as you can carry sufficient to place with the point and carefully rub the surface of the root upon which it is needed. In this way there is no danger of overdoing the medication. Then carefully dry the opening and surface of the tooth so that you can seal the pocket with steresol, or something similar. Steresol is a preparation recommended by Dr. M. L. Rhein, of New York.

If the pockets are sealed nature will have an opportunity to do some repairing, and for a time will not be interrupted by food particles, bacteria or saliva laden with toxic materials. If, after this treatment, the pocket does not heal it is because there is still some cause for irritation, due to deposits which have not been removed, necrosed bone or bacteria. Sometimes it is necessary to pack the pockets in order to assist the healing process, especially when considerable gum tissue or process has been destroyed. A very good and effective material for this purpose is iodoform cordine. The especial virtue of this is that it is already prepared to use, more easily manipulated and is much more nearly aseptic than would be a piece of cotton prepared on a broach by you at the chair. If a pocket needs to be packed it should be irrigated and repacked every day, allowing it to heal from the bottom toward the opening. If pus be present, do not begin to scale the roots until you have syringed out the pocket several times with some antiseptic, but never with pyrozone, for there is danger of its carrying the pus germs further into the surrounding tissues when it effervesces.

During treatment the importance of artificial stimulation to the tissues cannot be too strongly emphasized. In most cases of pyorrhea we have a condition of mal-occlusion in which case often there are some teeth which receive no use at all, consequently we have a stagnant circulation in the pulp and soft tissues. This can only be improved by artificial means, and the most effective method is by gum massage, vigorous brushing and by frequent hand polishing; the latter not only stimulates, but removes all source of irritation, so that the tissues have nothing to do but to recover.

The prophylaxis treatment should be given as frequently as the severity of the case demands until cured. In order to maintain a healthy state, the mouth and teeth must be kept free from accumulations which, if allowed to remain, will so irritate the tissues that a return of former conditions will be inevitable. But if given a healthy mouth it is a very easy matter to maintain such a condition, providing you will do your work faithfully.

How unfortunate it is that we must meet with such an awful mouth affection, especially in the practices of ethical dentists, but since we do we must treat it as we find it. However, we should plan for the future, and prevent in the coming generation such conditions as we see today. The Orthodontists are doing much to help us in that direction, and with the combined efforts of all, we should leave no cases of pyorrhea for our successors to treat. We must educate ourselves so that we can recognize the early stages of the disease.

If we can do this it will mean but little treatment in order to place the mouth in a healthy condition, and by keeping away all local irritants those tissues can be kept in a state of health. How much better is prevention than cure! The old adage, "A stitch in time saves nine," is very appropriate.

It is very frequently some dentist's fault that a patient has pyorrhea in its advanced stage, for usually we find that the patients who come for treatment late have been regular patients of some one for years, and that some one has either treated them inefficiently or has discouraged them completely with regard to the saving of their teeth.

It is not a difficult task to cure pyorrhea in its incipiency, and a healthy mouth is quite easily maintained such with your patient's co-operation. Pyorrhea *can* and *should be* prevented. It is your duty and it is mine. In 1880 Dr. George Mills, of Baltimore, made a prophecy, which is, I believe, being realized today and which will be even more applicable to the future. He says, in speaking of pyorrhea, "The time will come, and it is coming very fast, when men will take an interest in it and be anxious not only to take care of the cases, but they will come to observe that the results are so terrible in the mouth and upon the health of the tissues generally, that they will anticipate cases, knowing that prevention is better than cure. This time will come soon or late, just as stimulus is brought to bear upon our profession." Let us see that stimulus is *brought* to the profession in *heroic* doses. Those who are interested, do all you can. Pyorrhea *can* be cured and *kept cured*, and what is even of more importance, it *can be prevented*. Let us not only give relief to the suffering humanity, but let us hasten the time when suffering from Pyorrhea will not be known.

DISCUSSION.

Dr. N. S. Hoff: Mr. President, I should prefer very much that the ladies should conduct this discussion, since there are a number here who are specializing in this subject. I don't think I ever heard a better paper on this subject, in fact, I don't believe I ever heard so good a one. I find myself in such perfect harmony with everything that Dr. Rogers has said that I cannot offer any criticism; however, I don't think that is always necessary for us to criticise what other people are doing, unless we felt that they are in error. I don't seem to find any error in this paper. I am so well acquainted with the work of Dr. Rogers and know how well she does it that I feel diffident about discussing it. If I discuss it at all it will not be to criticise, but to emphasize some of the points she has brought out.

Why is it that so many dentists say pyorrhea cannot be cured, and speak of it as an incurable disease? I have noticed this tendency for some time and after studying the matter, I think I can throw a little light on the attitude of most practitioners toward this disease. The most obvious reason why we are saying pyorrhea cannot be cured is that we do not know how to treat it. This reason would be equivocal if it applied to the whole profession. Most practitioners take pride in doing their work well and think they have used all the intelligence and skill they possess in their efforts. to cure this disease; but it seems to me they fail to grasp the fundamental idea. Pyorrhea is not curable, it seems to me, by many because many dentists are trying to cure desparate and impossible cases. We cannot hope to restore to a normal condition the root of a tooth that has absolutely no bony support: it is useless to attempt it.

When I meet cases of that kind, unless it is the only tooth seriously affected and there is a chance to support it by splinting to adjoining teeth, I extract the teeth. There is little to be gained by subjecting a patient to a painful treatment if you know you cannot cure. I do not mean to say that I make it a positive rule to extract every tooth that seems hopelessly diseased. In many cases we find the teeth that are loose can be restored to good function by giving them an artificial support of some kind. In time such loose teeth as that will become set in the soft tissues and a certain amount of new bone tissue will form about them. These are not what I should call desparate cases. Desparate cases are such as preclude instrumentation or where it is almost impossible to scale the teeth and put them in any where near a normal condition, so far as cleanliness is concerned. If we can't get the teeth absolutely clean we shall get no cures. The sooner we learn to discriminate in these cases the sooner we can say that pyorrhea is curable. This is one point I wanted to emphasize.

The essayist brought out the point that thorough scaling is a fundamental principle. I don't think we can emphasize that any too strongly.

It is the surgical treatment that demands our best attention. The surgeon who leaves any infection or disease tissue in a wound has not made a thorough operation. The best surgeons of to-day, for instance in making operations involving any part of the body where the lymphatics are involved, anticipate the spread of disease and remove the lymphatics even in the healthy tissues, so as to take away all chance of the wound becoming reinfected. Now, I would not advocate that you go beyond the cause of the infection in treating pyorrhea and take away healthy tissues. But it is necessary for us to go to the full extent of the infection. In most of our operations we do not destroy all of the tissues that are hopelessly involved. As Dr. Rogers says, we do not have proper instruments; we do not have the training that we should have to enable us to know when we reach the limits of the infected areas. Some of the most successful men in treating this disease make this their fundamental principle, such men for instance as Dr. Younger, of Paris, and Dr. Good, of Chicago. The basis of their operations is a thorough removal of all the infected tissue. They succeed in getting admirable results, simply because they are acting upon this well know modern surgical principle. It is for us to follow if we expect to succeed.

My own method of performing these operations is not exactly the same that Dr. Younger and Dr. Good follows, but is based on the same surgical principle of radical cleanliness. I have not felt that it was necessary to go to the extremes to which Dr. Good and Dr. Younger do in operating, because I find that the least mutilation of the gums I do, the quicker the healing and the more effective the operation I do not wish to destroy any more of the gum than is absolutely required in order to reach the seat of infection. I especially value the gingival margins. I have bought every instrument on the market that I have seen that would enable me to perform the operation of scaling with as little injury as possible to the gums. My plan is to reach all of the deposit that is practicable through the natural openings into the sockets, but never going to the extent of tearing open the gum tissue at this point. I would rather open artificially into the gum at a point where it is not likely to be re-opened by disease, to the side of the root by a crucial incision, a position where it is more likely to withstand the influences which would cause the tissue to break down. This method will often facilitate the removal deposits, because it gives more direct access to the parts involved and leaves no detrimental scar. In whatever way we do this work, and each man will have his own method, the point is to do it thoroughly. If we can accomplish this part of the work thoroughly, and get the teeth in cleanly condition, and give them rigid support, there is no question in my mind at all but what this is an absolutely curable disease in a large majority of cases. It is in fact curable to such a large extent I think we can almost say it is absolutely curable in all cases. A case that is not curable would be where we are not able to treat it properly surgically, or the patient will not do his part of the work in keeping the mouth clean.

The question is always brought up as to the medicinal treatment and is one of importance. Many cases are so treated to-day exclusively. I have had a great deal of experience, as all of you have had, with medical treatment; but since I have taken up with this method of treatment I have almost entirely abandoned medicinal remedies, except as detergent disinfectants. If you can more thoroughly remove the deposit by solvent drugs or the necrotic bone wherever that is extensively diseased, all well and good; but drugs should not be used continuously nor should they be reapplied daily or oftener, as a great many think they should be, for you will destroy the healing tissues. The surgeon does not do this in his work. When he makes a wound he does not fill it full of irritating remedies to destroy the bacteria, but uses mild antiseptic washes. He simply washes and cleanses the parts and keeps them so. He wants them clean, but he is careful not to disturb the new granular tissues that will close up the wound.

When scaling a tooth we should not do this partly to-day and tell the patient to come again to-morrow to have it completed; do not keep opening the wound by continued scaling or probing. When a surgeon makes a wound he wants it to heal as soon as possible. If he is compelled to open it up again he considers it a bad piece of work. If you scale but one tooth at each sitting do it thoroughly and finally then you will have done your work on a scientific basis. Don't keep pricking at the gums all the time and thus opening wounds which run the risk of becoming re-infected. This, I believe is one of the great advantages of Dr. Younger's and Dr. Good's methods, they make but one operation. I believe if we shall adopt these surgical methods in connection with the prophylaxis treatment we shall all come to the conclusion that pyorrhea alveolaris is a curable disease. I can almost assure you that pyorrhea is curable if treated along these lines, and that every one can be successful with it.

DR. J. W. SWEETNAM: I have read the paper, but not heard it presented. Dr. Rogers has done admirably in presenting the matter and in substance it recommends thoroughness in operation. There is only one point in which I would differ. I use medicines, sulphate of copper has been a great help to me in the treatment of pyorrhea. Absolute thoroughness in doing the surgical work is the keynote to success. We all know when we leave any deposit on the roots of the teeth that the gums will not heal. The first thing is to remove all deposits from the roots and when this is accomplished, it makes very little difference what medication is used. I find it convenient to use sulphate of copper, for its stimulating and astringent reaction

Many make a mistake in undertaking more than can be thoroughly done at each sitting. If it be one tooth only, well and good, so far as you go do the work thoroughly. Even with our best efforts there will be cases sometimes that will not heal; then we have to go into the pockets deeper and see what the cause may be. We don't have to do this that will not heal; then we have to go into the pockets deepen and see what the cause may be. We don't have to do this very many times before we get results. There are lots of cases where I have failed in my first effort because I left some particle of deposit there and the gums failed to heal. In these cases after they are thoroughly healed I believe they are more immune to pyorrhea than they were in the first place It is a little harder to keep the teeth clean; patients must help, and they will if we give them the proper instructions. I have cases that have been under my observation twenty years with no re-occurrence. People apply to us with teeth that are practically lost. But where there is proper root to give the teeth the strength there is no such thing as not healing. And all there is to the healing is to keep them clean after the deposits are once thoroughly removed and the teeth polished.

DR. E. T. LOEFFLER: There is one point I wish to emphasize and perhaps draw your attention to a little more closely, and that is the education of the operator and the patient. I don't believe this work can be done by everybody, without an effort. In doing this work we must have oral prophylaxis. With a great many the first question is: I can't do that, as my patients won't pay for it, and we do only so much work as we feel the patient will pay for, consequently we do not get such results that we ought to. You and not your patient is then to blame for defective work. Patients will pay if they get value received; if you don't give them the value they will not pay. You can't do everything on your first trial. You can't expect to be successful at the treatment of pyorrhea without first learning how to do it. Learn how to handle your instruments, and then get your patients thoroughly interested, if you do not, your work will not be what it ought to be. Buy suitable instruments and learn how to use them and do the work thoroughly and get the co-operation of your patients.

Another point: I believe the systemic condition of every patient treated for pyorrhea ought to be looked after. If the system is as it ought to be we should not have this local manifestation at all. That looks like good sense. Thoroughly treat the local disease, that is right; but there is some disturbance behind it. If you can get the patient to do a little more exercise, drink plenty of water, it will help to bring about better and desired results; these things are absolutely necessary. One case I have in mind the patient suffered a great deal of pain, couldn't sleep nights. I recommended a little more out door exercise, plenty of fresh water to drink and was surprised to notice the beneficial results.

DR. WOOD: I think that this paper is the best I have ever heard on the subject, it is like a very good sermon. I should like time to re-read and to digest it without discussing it just now.

Oral prophylaxis as a treatment for pyorrhea does not mean simply cleaning teeth and substituting the name oral prophylaxis. It means persistent hard work intelligently applied, and no lazy man need think it an easy procedure.

Oral prophylaxis is one of the most important operations we can put our efforts into.

I believe there are as many teeth lost from lack of this treatment and more disorders brought about in the general system, than from neglected cavities in the teeth. The common practice in vogue of rattling around for fifteen minutes with dull scalers, or with engine polishers, is bad practice; it is only a bluff which lowers the moral fibre of the dentist and is not appreciated as worth anything by the patient; it is usually thrown in with other work without charge, and quite as often not done at all. The thorough removal of all deposits and polishing all surfaces is infinitely more scientific, more professional, more important and requires more skill than almost any other operation about the mouth. If done properly patients invariably realize that you have rendered them valuable service and are willing to pay for your time, the same as for other operations.

A man doing oral prophylaxis conscientiously and up to a high standard cannot help doing every other operation more carefully. I have proved to my own satisfaction that practically every case of pyorrhea can be cured to stay, if after all calculi has been removed the soft deposits are kept off by regular monthly treatments.

Any dentist who does not equip himself with proper instruments for oral prophylaxis treatment, and push it to the best of his ability, will most surely find himself sliding to the rear of the profession. Oral prophylaxis will do more to prevent commercialism in dentistry than all other influences combined.

Thorough prophylaxis methods require that we put our attention to the individual tooth in scaling and polishing. Persistent work on a single tooth until one is sure no deposits remain, and after having gone over every tooth in this same careful manner we shall feel a satisfaction in having accomlished something worthy our best efforts.

Each month will show your patients the particular places where they fail to keep off the soft deposits. Insist upon the use of good stiff brush and plenty of powder, floss silk and quil tooth pick.

Oral prophylaxis implies better fillings, better crowns, better bridges, better everything than any of us have ever done before as an outcome of pushing this part of our practice. Which one of us can or will do as good a piece of work in a badly kept mouth as he will in one well kept, that is clean and wholesome? Might as well expect a carpenter to do as good a piece of work in a pigpen as in a parlor.

There is no line of thought in dentistry more productive of good than that of prophylaxis. We cannot learn it all in a month, or a year, but it improves with every days work. When all colleges put in a chair of oral prophylaxis and the professor in charge insists upon it, and stays by the student until he has properly cleansed the mouth of clinical patients, this subject will be properly recognized by the profession generally. I believe we should fit ourselves in any branch of our work so that we have the utmost confidence in our ablilty to get results up-to-date and satisfactory to ourselves and patient. No branch requires any more skill than prophylaxis, and I advise any man who wishes to make a success of general dentistry to either go at it earnestly himself or get some dentist to do it for him. Then when an aggrivated case comes in he will not say there is no help for you, and send them to the extracting office. I know there are many people going about sick who would not be if they knew where to get their mouths put right.

Change of environment is what we must bring about to accomplish the cure of pyorrhea.

